# Development of an Electronic Alcohol Screening and Brief Intervention Program for Hospital Outpatients With Unhealthy Alcohol Use

**DOI:** 10.2196/resprot.2697

**Published:** 2013-09-20

**Authors:** Natalie A Johnson, Kypros Kypri, John Attia

**Affiliations:** ^1^School of Medicine and Public HealthFaculty of Health and MedicineThe University of NewcastleCallaghan, NSWAustralia; ^2^Hunter Medical Research InstituteNew Lambton, NSWAustralia; ^3^Department of General MedicineJohn Hunter HospitalNew Lambton, NSWAustralia

**Keywords:** alcohol, drinking, screening, brief intervention, hospital, outpatients, Internet

## Abstract

**Background:**

Alcohol screening and brief intervention is recommended for widespread implementation in health care systems, but it is not used routinely in most countries for a variety of reasons. Electronic screening and brief intervention (e-SBI), in which patients complete a Web-based questionnaire and are provided with personalized feedback on their drinking, is a promising alternative to practitioner delivered intervention, but its efficacy in the hospital outpatient setting has not been established.

**Objective:**

The objective of our study was to establish the feasibility of conducting a full-scale randomized controlled trial to determine whether e-SBI reduces alcohol consumption in hospital outpatients with hazardous or harmful drinking.

**Methods:**

The study was conducted in the outpatient department of a large public hospital in Newcastle (population 540,000), Australia. Adults with appointments at a broad range of medical and surgical outpatient clinics were invited to complete an e-SBI program on a laptop, and to report their impressions via a short questionnaire. Follow-up assessments were conducted 2-8 weeks later by email and post.

**Results:**

We approached 172 outpatients and 108/172 (62.8%) agreed to participate. Of the 106 patients capable of self-administering the e-SBI, 7/106 (6.6%) did not complete it (3 due to technical problems and 4 because they were called for their appointment), 15/106 (14.2%) indicated that they had not consumed any alcohol in the past 12 months, 43/106 (40.6%) screened negative for unhealthy alcohol use (scored less than 5 on the Alcohol Use Disorders Identification Test Consumption [AUDIT-C] questions), 33/106 (31.1%) screened positive for hazardous or harmful drinking (AUDIT-C score 5-9), and 8/106 (7.5%) screened positive for possible alcohol dependence (AUDIT-C score 10-12). Among the subgroup with hazardous or harmful drinking, 27/33 (82%) found the feedback on their drinking very, quite, or somewhat useful, 33/33 (100%) thought the intervention would appeal to most or some of the people who attend the service, and 22/30 (73%) completed the follow-up. We also found that some well established procedures used in trials of e-SBI in the primary care setting did not translate to the hospital outpatient setting (1) we experienced delays because the e-SBI program had to be developed and maintained by the health service’s information technology staff for security reasons, (2) recruiting patients as they left the reception desk was impractical because patients tended to arrive at the beginning of the clinics with few arrivals thereafter, and (3) use of a laptop in a fixed location resulted in some patients rushing through the e-SBI so they could return to their seat in the area they had been advised to wait in.

**Conclusions:**

e-SBI is acceptable to outpatients and with some adaptation to organizational and physical conditions, it is feasible to recruit and screen patients and to deliver the intervention without disrupting normal service provision. This suggests that e-SBI could be provided routinely in this important setting if shown to be efficacious.

## Introduction

### Alcohol Screening and Brief Intervention

Unhealthy alcohol use is a leading risk factor for premature death and disability globally [1]. Alcohol screening and brief intervention reduces unhealthy alcohol use in primary care patients who are not dependent on alcohol [2], and routine implementation in a variety of health care settings is recommended [3-5], but underutilized [6]. In Australia, for example, counseling or advice in relation to alcohol is provided at a rate of about .4 per 100 encounters in the primary care setting [7]. Provider-level barriers to the implementation of screening and brief intervention include time constraints, insufficient training, and the risk of damaging rapport with patients [8].

Electronic alcohol screening and brief intervention (e-SBI) is a promising alternative because it circumvents many provider-level barriers. Systematic reviews and meta-analyses of computer-delivered interventions have generally been positive [9-13], but most randomized controlled trials have studied computer literate young people with high rates of binge drinking [14], and most reviews have concluded there is a need for further research to establish the efficacy of e-SBI in other populations and settings [9,11,13,14]. Although there is solid evidence for the acceptability of e-SBI in primary health care [15] and the emergency department [16-18], and some evidence for efficacy in these settings [19-21], there appear to be no trials testing the acceptability, feasibility, or efficacy of e-SBI in the hospital outpatient setting aside from one trial of a brief computer-delivered intervention for alcohol use limited to pregnant women attending a hospital prenatal clinic [22]. Indeed, a recent systematic review of the effectiveness of drug and alcohol interventions offered opportunistically to patients aged 16 and older (excluding pregnant women) presenting to an acute hospital outpatient setting for any reason other than specifically for alcohol or illicit drug misuse treatment did not identify any trials testing the efficacy of e-SBI [23].

### Hospital Outpatient Settings

The hospital outpatient setting serves a large proportion of the population. In Australia, a country of 23 million people [24], 16.7 million service episodes were delivered in 2010-11 [25]. Although most research regarding the barriers to implementation of screening and brief intervention for unhealthy alcohol use by health care providers has been conducted in the primary care setting [8], the existing literature regarding alcohol interventions in the outpatient setting [26-31] suggests the barriers are similar. The overall aim of this study, therefore, was to determine the feasibility of conducting a full-scale randomized controlled trial (RCT) in the outpatient department of a large public hospital to determine whether e-SBI reduces alcohol consumption in hospital outpatients with hazardous drinking (a drinking pattern that increases the risk of harmful consequences for the user) [32], and harmful drinking (where damage to health is already occurring) [32]. The objectives of this study were to (1) adapt an existing e-SBI program for university students [33,34], to hospital outpatients, and ensure it complies with the health service’s information technology (IT) systems, (2) assess the feasibility of recruiting hospital outpatients with hazardous or harmful drinking, (3) test delivery in the outpatient waiting area, (4) gauge acceptability and identify any refinements needed, and (5) estimate likely follow-up rates.

## Methods

### Ethical Approval

Ethical approval for this study was obtained from the Hunter New England Human Research Ethics Committee (08/12/17/5.16) and the University of Newcastle Human Research Ethics Committee (H-2009-0332).

### Study Design and Setting

This single-arm feasibility study was conducted in the Ambulatory Care Center (outpatient department) at the John Hunter Hospital, a large public hospital located in Newcastle (population 540,000) [35], Australia. A broad range of medical and surgical outpatient services are provided by the Ambulatory Care Center including rehabilitation, transplant, vascular access, vascular surgery, pain management, oral and maxillofacial surgery, colorectal care, ears, nose and throat and head and neck surgery, general surgery, neurosurgery, opthalmology, orthopaedics and urology. Patients attending these clinics must have a written referral from their primary care provider and may bypass smaller hospitals in order to access specialist services provided by this large public hospital. Accordingly, patients may come from up to 500 kilometers away.

### Participants and Study Procedure

Adult (18 years of age or older) outpatients capable of self-administering the e-SBI instrument were eligible to participate. The recruitment process was modelled on research conducted by Kypri and colleagues in a New Zealand university student primary care service [36]. Research assistants located in the waiting area of the outpatient department were trained in the application of a study protocol stipulating they should invite the next patient leaving the reception desk to participate and to log consenting participants into the e-SBI program using a unique identifier. This identifier allowed us to link the paper-based data provided by participants with the data collected electronically and made it possible for participants who were interrupted (eg, were called for their appointment before completing the e-SBI) to continue the e-SBI rather than start again. As each participant finished, research staff would approach the next patient leaving the reception desk. The aim of this procedure was to minimize the risk that the research staff would exercise discretion in who to invite that could bias estimates of participation.

Eligible outpatients who gave written informed consent were invited to complete the e-SBI instrument and to provide feedback on their impressions of it via a short pen-and-paper questionnaire while waiting for their appointment. Participants were advised to stop the e-SBI if they were called for their appointment, so as not to interfere with normal service provision, but were asked to return to the waiting area to complete it before leaving the hospital.

Participants were followed-up using an adapted tailored design method [37] in which they received a letter reminding them about the study and advising that they would receive a brief follow-up questionnaire in the next few days. Although we sought ethical approval to include a supermarket voucher, an evidence-based strategy for increasing participation [38,39], we could only include a pen because the Hunter New England Human Research Ethics Committee had a policy of not approving “the offering of vouchers” as this was “regarded as an incentive and in breach of statement 2.2.10 of the National Statement on Ethical Conduct in Human Research (2007).”

Participants who reported consuming alcohol in the past 4 weeks (ie, those who might be eligible for inclusion in a trial) were followed-up in December 2010 (ie, 2-8 weeks after recruitment) regardless of the actual date of recruitment. This procedure was adopted in preference to rolling follow-up due to resource constraints. Participants who provided an email address received an email message with a link to the brief Web-based follow-up questionnaire, while those who did not provide an email address received a paper questionnaire by post. Up to three email/postal reminders were sent following the initial invitation to complete the follow-up surveys. Participants who did not respond to the initial and reminder emails/postal surveys were followed-up by telephone.

### e-SBI Program

The e-SBI program for hospital outpatients was based upon the Tertiary Health Research Intervention Via Email (THRIVE) program, which has been shown to reduce alcohol consumption among university students with hazardous or harmful drinking [33,34]. It comprised two parts (1) an assessment of drinking patterns, cognition, and alcohol-related harms, and (2) personalized feedback, including normative feedback, which some studies have shown to reduce alcohol consumption in heavy drinking students [40] and adult problem drinkers [41].

**Figure 1:**
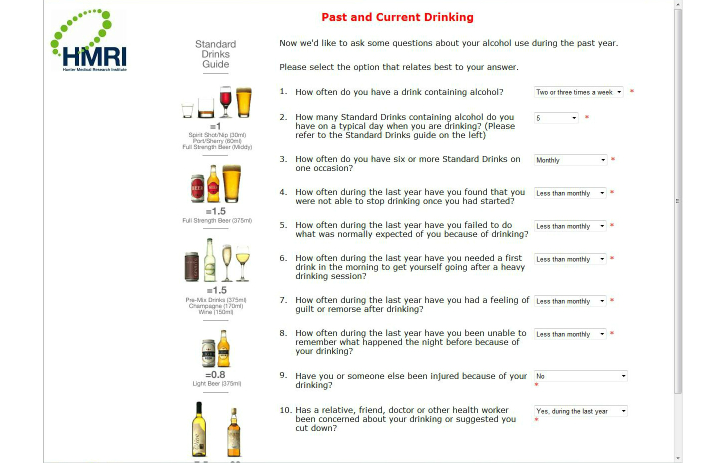
Screenshot from the pilot HOAP e-SBI program showing the AUDIT.

Page 1 provided a brief description of the Hospital Outpatient Alcohol Project (HOAP). Page 2 collected demographic data (gender, age, and postcode). Page 3 asked respondents if they had consumed alcohol in the last 12 months. Those who had not were sent to a “Thanks” page at this point, while those who had consumed alcohol proceeded to page 4. The Alcohol Use Disorders Identification Test (AUDIT) [42] comprised page 4 (Figure 1 shows this page). Page 5 asked questions concerning the largest number of standard drinks consumed in the patient’s heaviest drinking occasion in the last four weeks, the duration of that episode in hours, and the patient’s body weight, for the purpose of estimating their peak blood alcohol concentration (BAC). Page 6 comprised the 10-item Leeds Dependence Questionnaire (LDQ) [43], and page 7 comprised the 5-item History of Trauma Scale [44].

**Figure 2:**
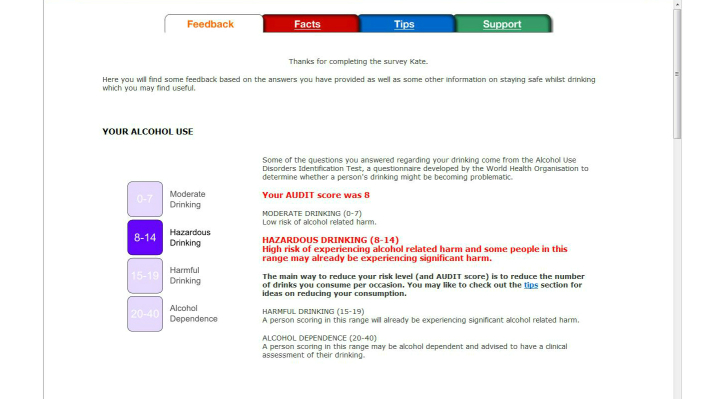
Screenshot from the pilot HOAP e-SBI program showing feedback regarding a hypothetical participant’s score on the AUDIT.

**Figure 3:**
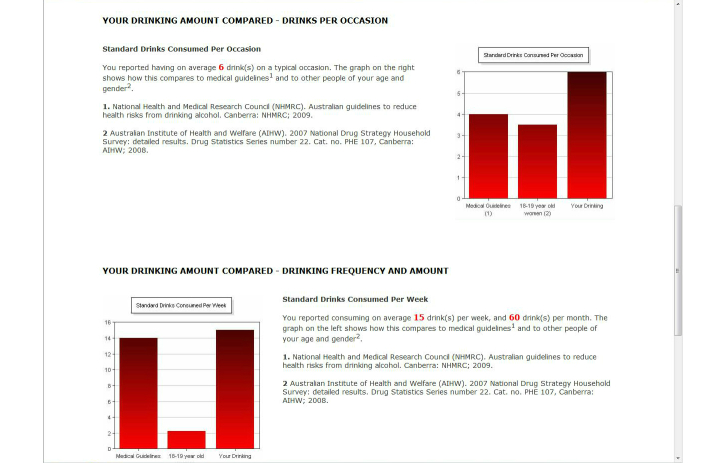
Screenshot from the pilot HOAP e-SBI program comparing a hypothetical participant’s (1) typical episodic consumption and (2) weekly consumption with (3) medical recommendations and (4) adults of the same age and gender.

All participants (ie, including those who screened negative for unhealthy alcohol use and those who screened positive for possible alcohol dependence) received (1) feedback on their AUDIT score and guidance on its meaning [42] (Figure 2 shows this page), (2) an estimate of the BAC for their heaviest drinking episode in the previous month with information on the behavioral and physiological sequelae of various BACs, and crash relative risk (not shown), (3) an estimate of their spending on alcohol per month (not shown), (4) a bar graph comparing their typical episodic consumption with medical recommendations [45] and that of adults of the same age and gender [46] (Figure 3 shows this page), (5) a bar graph comparing their weekly consumption with medical recommendations [45] and that of adults of the same age and gender [46] (Figure 3), and (6) their score on the LDQ with an explanation of the associated health risk and information about how to reduce that risk [43] (not shown). It is important to note that normative feedback via the bar charts was withheld when participants’ episodic or weekly consumption was lower than medical recommendations [45] in order to avoid the risk that participants might drink up to the norms [47]. In addition to the personalized feedback, three additional pages providing information about alcohol (eg, the consequences of unhealthy alcohol consumption), tips for reducing the risk of alcohol-related harm, and sources of support for drinking problems (eg, contact details for services available in the local health district) were provided. Participants had the option of emailing a copy of their personalized feedback to themselves. We chose not to provide a printed copy of the feedback because of concerns about confidentiality (eg, when printing is delayed, as a consequence of paper jams and so forth, people may see feedback other than their own).

### Outcomes

#### Recruitment

Participants in the proposed full-scale RCT will be screened for hazardous and harmful drinking using the AUDIT-Consumption (AUDIT-C) subscale [48]. This screening tool, which comprises the first three questions of the 10-item AUDIT and has similar specificity and sensitivity [48], will be used to minimize the risk of assessment effects [49] because administration of the full AUDIT alone has been shown to reduce self-reported drinking levels [50]. A minimum score of 5 points on the AUDIT-C will be used because it has high specificity while maintaining good sensitivity for identifying patients with hazardous or harmful drinking [48]. A maximum score of 9 will be used because the probability of alcohol dependence with a score above 9 is high [51], and these patients probably require more than brief intervention [52]. Thus although all participants in this study completed the AUDIT (ie, because they all received the intervention), the feasibility of recruiting outpatients with hazardous or harmful drinking was measured as the proportion of eligible consenting outpatients who scored 5-9 on the AUDIT-C.

#### Intervention Completion

The feasibility of delivering e-SBI in the waiting area of the outpatient department of a large, public hospital was measured as the proportion of participants who completed the e-SBI.

#### Acceptability of e-SBI

The acceptability of the e-SBI (eg, ease of completion, clarity of questions, privacy) was assessed using self-administered survey questions (1) immediately on completion of the e-SBI using a procedure described by Hallet et al [33], and (2) at follow-up. The questions and response options used at baseline and at follow-up are shown in the results section.

#### Retention

The feasibility of contacting participants to complete assessments of their drinking was measured as the proportion who returned the follow-up questionnaire comprising nine questions: three seeking information on alcohol consumption (“On how many days in the last 4 weeks did you drink alcohol?”, “On average, how many standard drinks did you have per drinking day?”, and “On how many days in the last 4 weeks did you have 6 or more standard drinks on one occasion?”), and six questions seeking feedback regarding the e-SBI program.

#### Data Analyses

Data were analyzed using STATA 11.1 (STATA Corporation, College Station, TX, USA). Descriptive statistics (frequencies and percentages for discrete variables and medians with interquartile ranges for continuous variables) were used to summarize the characteristics of study participants (gender, age group, and alcohol consumption) and outcomes related to recruitment, intervention completion, acceptability of e-SBI, and retention.

## Results

### e-SBI Program

The e-SBI program for hospital outpatients was essentially the same as the THRIVE program except for the addition of (1) the revised Australian drinking guidelines [45], (2) normative feedback regarding the amount of alcohol consumed by Australian men and women over 29 years of age [46], and (3) information regarding local sources of support for drinking (for example, contact details for services available in the local health district). Unplanned modifications associated with delivery of the intervention via the health service’s information systems included programming to recreate the e-SBI program by IT staff employed by the health service to ensure compliance with its systems, and the removal of links to external websites because of security concerns, such that participants could not be offered access to additional information on drinking guidelines, standard drink measures, and drink-driving legislation.

### Outcomes

#### Recruitment

**Figure 4:**
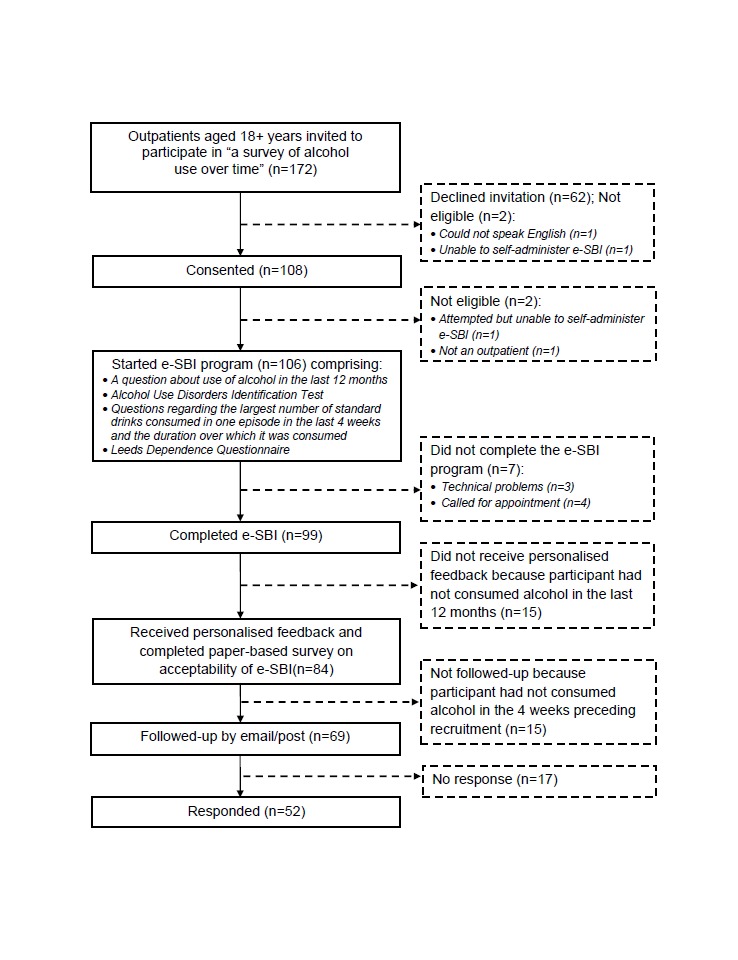
Flow of participants through pilot study.

Although research assistants were trained in the application of a study protocol stipulating they should invite the next patient leaving the reception desk to participate in the study, it quickly became apparent that this recruitment procedure was inefficient because patients arrived in large groups around the time that specific clinics opened, followed by long periods of time with very few arrivals. Our solution was to approach patients who occupied designated seats in rotation around the waiting area and it was often possible to approach all outpatients because of the long waiting times. Of the 172 outpatients we approached, 108/172 (62.8%) consented, 62/172 (36.0%) refused, and 2/172 (1.2%) were not eligible. Among those who consented, 2/108 (1.9%) were found to be ineligible and excluded (1 patient was unable to self-administer the e-SBI due to arthritis and the other person was not an outpatient). Among the 106 eligible consenting patients, 7/106 (6.6%) did not complete the e-SBI, 15/106 (14.2%) had not consumed any alcohol in the past 12 months, 43/106 (40.6%) screened negative for unhealthy alcohol use (scored less than 5 on the AUDIT-C), 33/106 (31.1%) screened positive for hazardous or harmful drinking (scored 5-9 on the AUDIT-C), and 8/106 (7.5%) screened positive for possible alcohol dependence (scored 10-12 on the AUDIT-C). Figure 4 shows the flow of participants through the study. The demographic characteristics of participants (n=99), and alcohol use among those who reported consuming alcohol in the past 12 months (n=84) are shown in Table 1.

#### Intervention Completion

Of the 106 eligible consenting outpatients, 99/106 (93.4%) completed the e-SBI program. Among the 7 noncompleters, 3/7 (43%) could not complete it due to technical problems, and 4/7 (57%) were called for their appointment before completing the program and did not return. In addition, because the laptop used to deliver the e-SBI was located 10-15 meters from some sections of the waiting area where outpatients had been advised to wait and from where they would be called for their appointment, we noticed that some participants were rushing through the program so they could return to the area they had been advised to wait in. This was a concern because it would reduce the efficacy of the intervention if participants did not read and absorb the feedback.

#### Acceptability of e-SBI

Feedback regarding the usability and acceptability of the program for all drinkers and the subgroup who screened positive for hazardous or harmful drinking is shown in Table 2.

#### Retention

Of the 69 participants who were invited to complete the follow-up assessment, 52/69 (75%) completed it. The follow-up rate among the subgroup with hazardous or harmful drinking was slightly lower, (22/30, 73%). Information obtained at follow-up is shown in Table 3.

#### Feasibility of Delivering e-SBI Using iPads

Due to concerns that arose during the pilot study regarding the usability of laptop computers, we returned to the outpatient waiting area six months later (June 2012) to assess the feasibility of using iPads. There were 9 patients (4/9, 44% male; 2/9, 22% aged 18-34 years; 4/9, 44% with an AUDIT-C score of 5-9) that agreed to participate. Although all were able to self-administer the e-SBI using the iPad, patients with larger fingers (mainly older men) would have found it easier if a stylus were available.

**Table 1:** Demographic characteristics and alcohol use of participants.

		Total	All drinkers	AUDIT-C Score		
		(n=99)	(n=84)	<5 (n=43)	5 - 9 (n=33)	>9 (n=8)
Male, n (%)		53 (54)	49 (58)	16 (37)	26 (79)	7 (88)
Age group, n (%)						
	18-34 years	33 (33)	29 (35)	11 (26)	14 (42)	4 (50)
	35-54 years	32 (32)	26 (31)	11 (26)	13 (39)	2 (25)
	55+ years	34 (34)	29 (35)	21 (49)	6 (18)	2 (25)
Access to email, n (%)		72 (73)	63 (75)	33 (77)	24 (73)	6 (75)
AUDIT score, median (25th and 75th percentiles)		-	5 (3, 12)	3 (1,4)	11 (7, 16)	19 (14.5, 27)
LDQ score, median (25th and 75th percentiles)		-	0 (0, 3)	0 (0, 0)	3 (0, 6)	5.5 (2, 11)
Consumed alcohol in the past 4 weeks, n (%)		-	69 (82)	32 (74)	30 (91)	7 (88)
Consumed more than 4 drinks on a single occasion at least once in the last 4 weeks, n (%)		-	41 (49)	5 (12)	29 (88)	7 (88)
Largest number of standard drinks consumed on a single occasion in the past 4 weeks, median (25th and 75th percentiles)		-	6 (3, 12)	2 (2, 4)	9 (7, 15)	20 (7, 24)

**Table 2:** Acceptability of e-SBI.

Question		All drinkers	AUDIT-C Score					
		(n=84) n (%)	<5 (n=43) n (%)	5-9 (n=33) n (%)		>9 (n=8) n (%)		
**How would you rate the level of computer competence required to complete the online survey?**								
	Very low	19 (23)	9 (21)		8 (24)		2 (25)	
	Low	29 (35)	15 (35)		13 (39)		1 (13)	
	Moderate	15 (18)	7 (16)		5 (15)		3 (38)	
	High	9 (11)	4 (9)		3 (9)		2 (25)	
	Very high	12 (14)	8 (19)		4 (12)		0 (0)	
**How hard was it to estimate how much or how often you drink?**								
	Very hard	1 (1.2)	0 (0)		0 (0)		1 (13)	
	Hard	5 (6)	1 (2)		2 (6)		2 (25)	
	Somewhat hard	16 (19)	2 (5)		12 (36)		2 (25)	
	Not hard at all	62 (74)	40 (93)		19 (58)		3 (38)	
**Did you respond honestly?**								
	All of the time	79 (95)	42 (98)		31 (94)		6 (75)	
	Most of the time	4 (5)	1 (2)		1 (3)		2 (25)	
	Some of the time	0 (0)	0 (0)		0 (0)		0 (0)	
	None of the time	0 (0)	0 (0)		0 (0)		0 (0)	
**How surprising was the feedback on your drinking?**								
	Very surprising	5 (6)	3 (7)		1 (3)		1 (13)	
	Quite surprising	14 (17)	3 (7)		9 (27)		2 (25)	
	Somewhat surprising	16 (19)	4 (9)		9 (27)		3 (38)	
	Not surprising at all	48 (57)	33 (77)		13 (39)		2 (25)	
**Was the feedback on your drinking useful?**								
	Very useful	21 (25)	11 (26)		6 (18)		4 (50)	
	Quite useful	20 (24)	7 (16)		11 (33)		2 (25)	
	Somewhat useful	25 (30)	13 (30)		10 (30)		2 (25)	
	Not useful at all	17 (20)	12 (28)		5 (15)		0 (0)	
**Will this affect how much you drink in the future?**								
	Yes	11 (13)	5 (12)		6 (18)		0 (0)	
	No	51 (61)	32 (74)		16 (48)		3 (38)	
	Possibly	21 (25)	6 (14)		10 (30)		5 (63)	
**Did the amount of privacy you had concern you? (Did it affect your answers?)**								
	Yes, all of the time	1 (1)	1 (2)		0 (0)		0 (0)	
	Yes, most of the time	0 (0)	0 (0)		0 (0)		0 (0)	
	Yes, some of the time	1(1)	1 (2)		0 (0)		0 (0)	
	No, none of the time	81 (96)	41 (95)		32 (97)		8 (100)	
**Were questions clear?**								
	Yes, all of the time	71 (85)	37 (86)		26 (79)		8 (100)	
	Yes, most of the time	11 (13)	6 (14)		5 (15)		0 (0)	
	Yes, some of the time	1 (1)	0 (0)		1 (3)		0 (0)	
	No, none of the time	0 (0)	0 (0)		0 (0)		0 (0)	
**Was the font size large enough to read?**								
	Yes	79 (94)	41 (95)		30 (91)		8 (100)	
	No	4 (5)	2 (5)		2 (6)		0 (0)	
**Do you think this online intervention will appeal to people who attend this service?**								
	Yes, all of them	12 (14)	11 (26)		0 (0)		1 (13)	
	Yes, most of them	41 (49)	22 (51)		15 (45)		4 (50)	
	Only some of them	30 (36)	10 (23)		17 (52)		3 (38)	
	None of them	0 (0)	0 (0)		0 (0)		0 (0)	

**Table 3:** Alcohol consumption and acceptability of e-SBI at follow-up.

		All drinkers	AUDIT-C Score		
		(n=52)	<5 (n=26)	5-9 (n=22)	>9 (n=4)
Number of days consumed alcohol in the past 4 weeks, median (25^th^and 75^th^percentile)		9.5 (3, 20)	4 (2, 10)	11.5 (5, 20)	23 (13.5, 28)
Number of standard drinks per typical drinking occasion in the past 4 weeks, median (25^th^and 75^th^percentile)		2 (1, 4)	2 (1, 2)	3 (2, 6)	9 (7, 10)
Number of times more than 6 standard drinks were consumed in past 4 weeks, median (25^th^and 75^th^percentile)		0 (0, 3)	0 (0, 0)	2 (0, 4)	10.5 (5, 15)
**I found the questionnaire easy to complete, n (%)**					
	No	3 (6)	2 (8)	0 (0)	1 (25)
	Yes	48 (94)	24 (92)	21 (100)	3 (75)
**I found the feedback on my drinking useful, n (%)**					
	No	7 (14)	4 (15)	3 (14)	0 (0)
	Yes	33 (65)	17 (65)	13 (62)	3 (75)
	I did not receive this feedback but would like to receive it	6 (12)	4 (15)	2 (10)	0 (0)
	I did not receive this feedback and am not interested in receiving it	5 (10)	1 (4)	3 (14)	1 (25)
**The feedback I received on my drinking included comparisons of my drinking with the average drinking levels of others the same age and gender as me. The averages presented were, n (%):**					
	About what I expected	23 (46)	11 (42)	9 (45)	3 (75)
	Higher than I expected	5 (10)	3 (12)	1 (5)	1 (25)
	Lower than I expected	3 (6)	1 (4)	2 (10)	0 (0)
	I had no idea what the average was	10 (20)	7 (27)	3 (15)	0 (0)
	I did not receive this feedback but would like to receive it	5 (10)	3 (12)	2 (10)	0 (0)
	I did not receive this feedback and am not interested in receiving it	4 (8)	1 (4)	3 (15)	0 (0)
**As a consequence of receiving the feedback the amount of alcohol I consume has, n (%):**					
	Not changed	39 (81)	23 (92)	13 (68)	3 (75)
	Decreased	9 (19)	2 (8)	6 (32)	1 (25)
	Increased	0 (0)	0 (0)	0 (0)	0 (0)
**I have sought support to reduce my drinking as a consequence of receiving the feedback, n (%)**					
	No	37 (77)	19 (76)	15 (79)	3 (75)
	Yes	11 (23)	6 (24)	4 (21)	1 (25)
**I would recommend this program to a friend if I were concerned about how much they were drinking? n (%)**					
	No	77 (34)	8 (31)	6 (30)	3 (75)
	Yes	23 (66)	18 (69)	14 (70)	1 (25)

## Discussion

### Principal Results

Our results show that e-SBI is acceptable to hospital outpatients and that it is possible to recruit, screen, and deliver e-SBI in the hospital outpatient setting without disrupting normal service provision. Almost two-thirds (108/172, 62.8%) of the patients we approached consented, almost two in five adults (41/106, 38.7%) reported unhealthy alcohol use (compared with one in five adults aged 18 years and over in the general Australian population) [53], and almost three-quarters (22/30, 73%) of the hazardous and harmful drinkers (ie, those who would be eligible for inclusion in a trial of the efficacy of e-SBI) completed the follow-up assessment.

In addition to obtaining estimates of the consent rate, the proportion that would be eligible for inclusion in a full-scale RCT, and the likely response at follow-up, we discovered that some well established procedures used in trials of e-SBI in the primary care setting did not translate to the hospital outpatient setting. First, we could not utilize the services of IT staff who had been involved in the development of the THRIVE program because of the health service’s requirement that the program use a particular programming language. This reliance on personnel employed by another organization who had other priorities delayed the project considerably. Second, the health service did not allow the inclusion of links to external websites for security reasons. While suboptimal, this is not a major concern because previous analyses of the Web pages accessed by more than 1000 users of the THRIVE instrument showed the e-SBI was efficacious [34] even though very few (64/1251, 0.05%) participants accessed the hyperlinks to external websites [33]. Third, it was not efficient to recruit patients as they left the reception desk and an alternative strategy had to be developed. Fourth, delivery of the e-SBI using a laptop in a fixed location, because of the need to connect to the hospital’s intranet, seemed problematic for participants who had to move 10-15 meters away from the area they had been advised to wait in. Delivery of the program via iPads, connected wirelessly to a server located in a room behind the reception desk, solved this problem as patients could participate without leaving their seats. This closed system, in addition to removing the problem of patients rushing through the e-SBI so they could return to their seat, had the advantage of returning control over the development and maintenance of the program to the research team (ie, we could employ IT staff familiar with our research to develop the program, and ensure a timely response to problems as they arose). Fifth, as some patients were called for their appointment before completing the program, we believe implementation of a trial would be facilitated by obtaining permission to send a hyperlink to the e-SBI program to participants who are interrupted in preference to asking them to return to complete it after their appointment. Finally, because undecipherable handwritten email addresses impeded follow-up contact, we recommend that patients be asked to enter their email addresses electronically, with the possibility of validating addresses also worth considering.

### Limitations

Limitations of the study include the short follow-up, attrition, and the small number of participants who completed the e-SBI using an iPad. The loss-to-follow-up is a concern because attrition reduces the effective sample size and can bias effect estimates [54]. We were prevented by an Ethics Committee policy from employing a key evidence-based strategy for increasing questionnaire completion rates [38], namely, the use of token incentives. Use of such strategies would probably increase the follow-up rate among hazardous and harmful drinkers from the 22/30 (73%) observed here to 24/30 (80%) or higher, putting it into an acceptable range for a trial of this type. Our finding that most participants could easily self-administer the e-SBI using an iPad is consistent with the findings of a Canadian study in which most (318/348, 91.4%) patients indicated that the iPad was easy to use [55]. Our observation that some patients would benefit from having a stylus available is also consistent with the Canadian study, which reported “some of the older users…seemed to struggle to adapt to the sensitivity and responsiveness of the touch screen” [55]. As 27/99 (27%) of our study participants did not have access to email (ie, did not have the option of emailing a copy of the feedback to themselves to read and reflect upon later), our decision not to provide printed copies of the personalized feedback may also be a limitation. Accordingly, we plan to devise a new process for generating and sending a printed copy of the personalized feedback to participants in the proposed RCT.

### Strengths

Strengths of the study include the use of an intervention informed by more than a decade of research on the development and evaluation of e-SBI in university students [34,36,56,57], the inclusion of a respected senior clinician with strong links to the health service and the university on the research team, and the mixed-mode follow-up. The fact that few modifications to the e-SBI program were required on the basis of feedback from our pilot study participants supports our view that the extensive developmental work on the THRIVE instrument and its predecessors [33] has produced an instrument that is acceptable to a wide range of people in a variety of settings and is a strength of this study. Inclusion of a senior clinician on the research team facilitated access to the outpatient department and gave us a “voice” when progress on the e-SBI stalled because of IT problems. Although the use of mixed contact modes for follow-up may be considered a limitation, it was a deliberate decision to facilitate inclusivity of people from households with lower incomes where home Internet access is less common [58]. Excluding patients without such access would make the findings less generalizable to poorer people; potentially further increasing health disparities [59]. In addition, offering different response modes sequentially-Web first with mail as the final contact-has recently been shown to improve response rates [39]. Since randomization protects against bias by modality of follow-up, especially where a large number of individuals is randomized, we intend to utilize mixed contact modes for follow-up in the proposed RCT.

### Conclusions

We obtained estimates of the consent rate, proportion with hazardous or harmful drinking, and response at follow-up, which are essential to the design of a full-scale RCT to determine whether e-SBI reduces hazardous or harmful drinking in hospital outpatients. In addition, our study demonstrated that e-SBI is acceptable to hospital outpatients with hazardous or harmful drinking and, given the feasibility of recruiting and screening patients, and of delivering the intervention without disrupting normal service provision, that it could be provided routinely in this important setting.

## Abbreviations

AUDIT: Alcohol Use Disorders Identification Test
AUDIT-C: Alcohol Use Disorders Identification Test Consumption subscale
BAC: blood alcohol concentration
e-SBI: electronic alcohol screening and brief intervention
IT: information technology
LDQ: Leeds Dependence Questionnaire
